# Is Curcumine Useful in the Treatment and Prevention of the Tendinopathy and Myotendinous Junction Injury? A Scoping Review

**DOI:** 10.3390/nu15020384

**Published:** 2023-01-12

**Authors:** Alfredo Córdova, Franchek Drobnic, David Noriega-González, Alberto Caballero-García, Enrique Roche, Melchor Alvarez-Mon

**Affiliations:** 1 Department of Biochemistry, Molecular Biology and Physiology, Faculty of Health Sciences, GIR Physical Exercise and Aging, University of Valladolid, Campus Duques de Soria, 42004 Soria, Spain; 2 Medical Department, Wolverhampton Wanderers FC, Wolverhampton WV1 4QR, UK; 3 Department of Surgery, Ophthalmology, Otorhinolaryngology and Physiotherapy, Faculty of Medicine, Hospital Clínico Universitario de Valladolid, 47003 Valladolid, Spain; 4 Department of Anatomy and Radiology, Faculty of Health Sciences, GIR Physical Exercise and Aging, University of Valladolid, Campus Los Pajaritos, 42004 Soria, Spain; 5 CIBER Fisiopatología de la Obesidad y Nutrición (CIBEROBN), Instituto de Salud Carlos III (ISCIII), 28029 Madrid, Spain; 6 Department of Applied Biology-Nutrition, Institute of Bioengineering, University Miguel Hernández, 03202 Elche, Spain; 7 Alicante Institute for Health and Biomedical Research (ISABIAL), 03010 Alicante, Spain; 8 Department of Medicine and Medical Specialty, Faculty of Medicine and Health Sciences, University of Alcalá, 28871 Alcalá de Henares, Spain; 9 Immune System Diseases-Rheumatology and Oncology Service, University Hospital “Príncipe de Asturias”, 28871 Alcalá de Henares, Spain

**Keywords:** cytokines, curcumine, exercise, inflammation, nutrition, tendinopathies

## Abstract

Physical activity in general and sports in particular, is a mechanism that produces stress and generates great force in the tendon and in the muscle-tendon unit, which increases the risk of injury (tendinopathies). Eccentric and repetitive contraction of the muscle precipitates persistent microtraumatism in the tendon unit. In the development of tendinopathies, the cellular process includes inflammation, apoptosis, vascular, and neuronal changes. Currently, treatments with oral supplements are frequently used. Curcumin seems to preserve, and even repair, damaged tendons. In this systematic review, we focus more especially on the benefits of curcumin. The biological actions of curcumin are diverse, but act around three systems: (a) inflammatory, (b) nuclear factor B (NF-κB) related apoptosis pathways, and (c) oxidative stress systems. A bibliographic search is conducted under the guidelines of the Preferred Reporting Items for Systematic Reviews and Meta-Analyses (PRISMA) as a basis for reporting reliable systematic reviews to perform a Scoping review. After analysing the manuscripts, we can conclude that curcumin is a product that demonstrates a significant biological antialgic, anti-inflammatory, and antioxidant power. Therefore, supplementation has a positive effect on the inflammatory and regenerative response in tendinopathies. In addition, curcumin decreases and modulates the cell infiltration, activation, and maturation of leukocytes, as well as the production of pro-inflammatory mediators at the site of inflammation.

## 1. Introduction

Tendinopathies are frequent problems in TraumatologyServices, being approximately 30% of the pathology in the locomotor apparatus. Inflammatory or degenerative tendinopathies occur in the general population and especially in older people. Various conservative strategies have been proposed to treat tendinopathies, including the use of dietary supplements, to promote physiological turnover of tendon tissue, in order to prevent inflammation and de-generation [1,2]. 

Physical activity in general, and sport in particular, is a stress mechanism that generates a great force on the tendon and in the muscle-tendon junction that increases the risk of tendon injuries [3]. Many anatomical areas can be affected, depending on the type of activity or sports discipline. Particular interest is centered in Achilles tendinopathy, patellar tendinopathy, and epicondylitis, which have a high incidence in athletes. In addition to sports discipline, the development of tendinopathy can result from modifiable and non-modifiable risk factors, such as age and gender. In this context, adolescents seem to be less affected by tendinopathies compared to adults [4].

The myotendinous junction (MTJ) plays a key role in this group of pathologies, because MTJ is the structure where muscle fibers interact with tendons [5]. The MTJ is structurally specialized to transmit force [6]. Despite this, force applied to the MTJ can be so extensive that can result in strain injuries, especially during eccentric loading [7]. For this reason, these are among the most frequent injuries in many popular sports [8,9]. The highly folded muscle membrane at the MTJ increases the contact area between muscle and tendon and potentially the load tolerance of the MTJ. Upon strong contraction of the sarcomeres, there is great tension of the muscle provided by the tendon which can generate injury by disabling the forces. In experiments in induced strain injuries in animal muscles, in which the muscles are pulled while being stimulated to contract, it has been observed that a disruption occurs in the distal part of the muscle fibers or in the basal lamina between the muscle fiber and the tendon [10]. This observation confirms that the connection between the muscle fibers and the tendon is a weak area [11].

Patients with tendinopathy often present persistent symptoms of mild to moderate intensity, leading to a decrease in physical activity and consequently a negative change in the quality of life. The primary cause of tendon degeneration seems to be due to mechanical overload followed by an inadequate recovery, leading to a failure of traction and tension of the collagen fibres within the tendon. Repetitive contraction of the muscle precipitates a persistent microtrauma in tendons. This situation lacks an adequate recovery due to the slow collagen turnover. In the long term, this can lead to significant degenerative changes that present clinical symptoms after clinical examination [12,13,14,15].

In tendinopathies, both macro and microscopic, tendon collagen fibres appear thinner, deorganised, and with associated fibrotic changes. Injured tendons have a higher concentration of type III collagen in contrast to healthy tendon tissue, which is mainly composed of type I collagen (COL-I) [15,16]. The cellular process in the tendon includes inflammation, apoptosis, vascular, and neuronal changes. In addition, specific factors must be considered in the development of tendinopathy, including gender, previous diseases or injuries, age, and flexibility [17].

Different molecules can play a negative role in the biology of tendon tissue and represent a risk factor in developing tendinopathy. Corticosteroids, antibiotics such as Quinolone, aromatase inhibitors, and statins as inhibitors of β-Hydroxy β-methylglutaryl-CoA (HMG-CoA) reductase are the drugs most frequently associated with altered tendon properties [18].

On the other hand, tendon pathologies can be the first clinical sign in various metabolic diseases, such as gouty arthritis and hypercholesterolemia. These pathologies present a high degree of inflammation that may be responsible for tendon degeneration. In patients affected by diabetes, glycation end products (including collagen) impair the biological and mechanical functions of tendons and ligaments [19,20,21].

Oral treatments with glucosamine, chondroitin sulphate, vitamin C, hydrolysed COL-I, L-arginine, curcumin (present report), among others, can help to preserve, or even repair, damaged tendons [22,23,24,25,26,27,28]. These supplements are not subject to rigorous pharmaceutical controls. However, some of these products have received permission to be sold as medicines in many countries. Recently, nutraceutical products have become more and more popular as they seem to be efficient in the prevention and healing processes of tendon injuries. 

The present review is focused on curcumin supplementation as a useful agent in the treatment of tendinopathies. The aim is to summarise current knowledge and areas of ongoing research in the treatment of tendinopathies using curcumin supplementation.

Tendons have an active metabolic activity mainly focused on energy production and the synthesis of extracellular matrix (ECM) and collagen. This protein is the main component of the tendon (30% of the total tendon mass). The remaining 70% is water. Type I collagen represents about 65–80% and elastin is 2% of the dry mass of tendons [29,30].

Collagen molecules are arranged in fibres especially designed to transmit forces between muscle and bone. Tenocytes and tenoblasts are cells located between the collagen fibres along the longitudinal axis of the tendon [31].

Tendinitis (inflammation of the tendon), tendon rupture, tenosynovitis (inflammation of the tendon lining), and calcifications, are the most common tendon affections. Along with this review, we will only focus on those characterised by an inflammatory process. In this respect, several classifications of tendinopathies have been established from a lesional or anatomopathological point of view. Nevertheless, we have chosen a simple classification centered on reactive and degenerative tendinopathy [3,32].

(a) Reactive tendinopathy (mechanical) is usually the consequence of an acute overload on the tendon due to tension or compression. In this type, the immune system plays a key role in tissue regeneration and maintenance processes. In this event, tendinopathy sometimes coincides with an episode of a cold or after oral surgery. From a microscopic point of view, cellular hyperactivity of the tendon can be observed after physical stress in reactive tendinopathy. The number of tendon cells increases greatly, the cells become rounded and the tissue attracts water due to the presence of hydrophilic molecules (i.e., proteoglycans). These machromolecules (proteoglycans and glycosaminoglycans) are important for water retention and improve the biomechanical properties (elasticity) in the tendon against shear and compression forces [33].
(b) Degenerative tendinopathy is characterised by a progression of ECM and collagen disorganisation. Cellular damage is accompanied by alterations in tendon vascularization, being unable to withstand any tensional stress, and evolving to be mechanically useless. In addition, the tissue begins to produce pain-related substances and activates the peripheral nerve, causing hypersensitivity [34].

### 1.1. The Inflammatory Process in Tendinopathies

Some years ago, tendinopathy was thought to be a degenerative process However, the development of tendinopathy is preceded by an inflammatory process. Several subtypes of immune cells and inflammatory mediators play a key role in the initiation and progression of tendinopathy [35].

During the progression of tendinopathies, both collagen in ECM and tenocytes are affected [36]. Repetitive mechanical overload and hypoxic injury, together with intracellular stress factors, are the main causes of tendinopathy, leading to the presence of elevated inflammatory markers [37]. 

High tendon requirements are accompanied by high temperatures within the tendon, which can increase apoptotic phenomena. Heat stress and hypoxia can lead to the overexpression of molecules such as matrix metalloproteinases (MMP) that promote ECM degradation and overproduction of inflammatory cytokines. In addition, the effect of hypoxia is associated with increased expression of vascular endothelial growth factor (VEGF). When overload exceeds the capacity for recovery, a pathogenic sequence leads to tendinopathy in the MTJ [11,16].

Tendon and MTJ injuries are accompanied by inflammation, with the expression of inflammatory mediators including proinflammatory and anti-inflammatory cytokines and growth factors produced by tenocytes, such as tumor necrosis factor-α (TNF-α), interleukin (IL)-1β, IL-6, IL-10, VEGF, transforming growth factor-β (TGF- β), cyclooxygenase-2 (COX-2), and prostaglandin E2 (PGE2) [37,38,39,40,41,42]. TNF-α and IL-1β promote the production of IL-6, a cytokine with a dual role in inflammation, tissue injury, and repair [43]. In this context, IL-6 seems to play a central role in acute stress response by initiating anti-inflammatory and restorative processes [43]. However, when the damage presents a significant pathological component, IL-6 induces an inflammatory response [39]. In this line, IL-6 is not only involved in inflammation but it is implicated as well in the early stages of tendon healing by promoting increased expression of COL1A1 (type I collagen-α1 chain) in tendons. The COL1A1 gene codes part of the type I collagen molecule [39,43].

In the acute phase of the inflammatory response, tenocytes synthesize proinflammatory cytokines, with a positive feedback mechanism that causes edema and hyperemia [16,44]. In addition, cytokines and immunomodulatory factors provoke cell proliferation, pain, and degradation of ECM with necrosis. ECM dynamics are instrumental in restructuring tissue architecture. The ECM constantly undergoes remodelling processes with degradation or modifications [16,38,45,46,47,48,49].

During the acute phase of the inflammatory process, there is an imbalance between proinflammatory factors, with ECM degradation; and protective factors, leading to pain, due to the release of substance P, glutamate, COX-2, PGE2, and PGE3 [50,51]. TNF-α, IL-1β, VEGF, and low levels of COL-I potentiate the inflammatory state and favour increased MMP activity, accompanied by a degenerative response and increased risk of tendon rupture [16].

The MMPs are a family of proteolytic enzymes that have the ability to degrade the components of the ECM network. MMPs are also factors that promote the healing process. The MMPs involved in tendon damage are MMP 1, 2, 3, 8, 9, 13, 14, 19, and 38, and their activation results from an imbalance with their inhibitors, the TIMPs (tissue inhibitors of MMPs). Therefore, tendon homeostasis is the result of a continuous remodelling process mediated by MMPs and TIMPs with the apposition ECM and production of COL-I [3,49,52,53].

On the other hand, VEGF (responsible for neoangiogenesis) is released after hypoxic stress in the early stages of tendinopathy. VEGF favors inflammatory mediators to gain access to the injury site. VEGF, together with cytokines such as IL-6 and IL-21R, is able to stimulate the expression of MMPs. This process is associated with ECM degradation and inhibition of TIMP expression in several cell types such as tenocytes, fibroblasts, chondrocytes, and endothelial cells [38]. The VEGF, together with other growth factors secreted by inflammatory cells, such as epidermal growth factor (EGF), fibroblast growth factor (FGF), TGF-β and insulin-like growth factor-I (IGF-I), promote tenocyte proliferation and the release of COX-2, PGE2, and prostacyclins. These last messengers are involved in the onset of pain in acute tendinopathy [38]. From the cytokines, the IL-21 is a pleiotropic cytokine, belonging to the common γ-chain cytokine family, mainly produced by T follicular helper (Tfh) cells, Th17 cells, NKT cells, and neutrophils [54]. IL-1β is upregulated in the synovial membrane, enhancing inflammatory reactions in injured joints. IL-1β contributes as well to the onset of proliferative and degenerative changes in the tendon [53]. The actions of this cytokine include local effects on the endothelium, induction of systemic acute phase reactions, and deleterious effects on tenocytes. IL-1β acts through its specific receptor on tenocytes, which after binding, can activate numerous signaling pathways, including those modulated by mitogen-activated protein kinases (MAPKs). These proteins play a key role in intracellular signal transduction, allowing the cell to integrate different extracellular stimuli activated by growth and stress factors. IL-1β also induces inflammatory mediators such as COX-2, PGE2, and MMPs [53]. Otherwise, IL-1β increases the expression of PGE synthase, which catalyses the final step in the conversion of PGH2 to PGE2. In addition, IL-1β stimulates the expression of the EP4 receptor (G protein-coupled PGE2 receptor) in tendon cells. Specific upregulation of EP4 receptors on tenocytes by inflammatory cytokines may enhance inflammatory signaling pathways. This ultimately leads to tendon matrix degradation and thus tendinopathy. On the other hand, IL-1β reduces the expression of COL-I at the mRNA level in human tissues, which may lead to less ECM deposition during tendinopathies [53]. IL-1α, on the other hand, affects inflammatory and immune responses, angiogenesis, and cell formation processes [37].

The healing process of tendon ruptures is mediated primarily by MMPs and thrombosponin motif metalloproteinases (ADAMTS) and their tissue inhibitors (TIMPs) [52,55,56].

It has been suggested that nitric oxide (NO) may play a role in the recovery process of tendinopathies. NO is synthesised by nitric oxide synthetases (NOS). There are four isoforms of the NOS enzymes: neuronal, endothelial, mitochondrial, and inducible (iNOS). In this context, iNOS is an isoform that can be induced by pro-inflammatory cytokines [57,58]. It has been suggested that NO may promote increased collagen synthesis [59], which would also be important in cases of tendon scarring. Semis et al. [60] have observed that after collagenase injection into rat tendons, malondialdehyde (MDA) (an indicator of lipid peroxidation) increases. These authors suggest that this fact contributes to the formation of tendinopathy by affecting tendon homeostasis and triggering inflammatory and apoptotic processes in the tendon tissue.

### 1.2. Therapeutic Management of Tendinopathies

Conservative treatment using eccentric exercises (with or without pain) has been shown to provide very good results at short and medium term in patients with tendi-nosis. This approach is associated with a decrease in fragility and thinning, favouring hypertrophy and a more normal appearance of the tendon structure. However, there is no scientific evidence of histological adaptations caused by excentric training [61].

Actually, different physical (exercise), physiotherapeutic (cryotherapy, electro-therapy, etc.), pharmacological (analgesics and antiinflammatory drugs), biological treatments (platelet-rich plasma (PRP), stem cells and autologous blood injections) are currently used [62].

### 1.3. Nutritional Approach to Tendinopathy

The fundamental therapeutic approach to tendinopathies in medicine is based on the use of pharmacological products and physical and manual techniques [63]. The role of nutrition as an effective tool to prevent and support the treatment of certain pathologies is gaining increased recognision. The use of nutritional supplements has to be considered, due to their metabolic effects on injured tissue in repair processes [2] and the relative absence of side effects.

Among the vitamins, the most accumulated evidence in tendon repair processes corresponds to vitamins C and D. Vitamin C is instrumental due to its antioxidant properties and its action as a coenzyme in collagen synthesis through the hydroxyla-tion of proline and lysine to hydroxyproline and hydroxylysinerespectively. Both hy-droxylated amino acids are essential for collagen structure and tendon function [28,64,65]. Thus, vitamin C deficiency is associated with a decrease in the synthesis of procollagen, due to a lower hydroxylation of proline and lysine residues [66,67].

Vitamin D is another nutritional element that has recently been gaining im-portance. Vitamin D is considered to act directly on collagen synthesis by tenocytes. When vitamin D was added to fibroblast culture media obtained from human tendons, a dose-dependent anabolic effect was demonstrated, with a progressive increase in COL-I mRNA levels. A decrease in ROS (reactive oxygen species) and MMP expression was also observed. These facts demonstrate that vitamin D has a positive effect on tendon tissue, and its deficiency could be a limiting factor for collagen synthesis as well as an increased oxidative stress [68,69]. Furthermore, in animal models, it has been observed that an increase in vitamin D, compared to deficient animals, improves car-tilage organisation and strengthens postsurgical tendon to bone scars [70]. Therefore, there is a positive correlation between vitamin D levels and the strength of tendon healing [71].

Amino acids have also been proposed as supportive nutrients in the treatment of tendinopathies due their key role in protein synthesis. A positive relationship has been observed between leucine and collagen synthesis. Leucine, an essential amino acid, causes increased levels of hydroxyproline which, in addition to being a fundamental component of collagen, plays an essential role in fiber stability [72]. Glycine is also a key element in collagen synthesis. A diet containing 5% glycine correlates with an in-crease in the synthesis of hydroxyproline and glycosaminoglycans and thereby, a greater capacity for synthesis of collagen molecules [73].

In this nutritional context, curcumin is becoming increasingly important in the process of connective tissue recovery, in particular after tendinopathy and MTJ.

### 1.4. Curcumin as a Therapeutic Adjuvant in Tendinopathy

*Curcuma longa* is a rhizomatous perennial herbaceous plant of the Zingiberaceae family, native to Southeast Asia, rich in bioactive molecules that have numerous health and therapeutic properties, among which the most researched are anti-inflammatory [74].

The biological actions of curcumin are diverse, but they are locked around three systems: (a) inflammatory, (b) nuclear factor κB (NF-κB) related apoptosis pathways, and (c) oxidative stress systems [75].

Curcumin and metabolic derivatives are potent antioxidants. This is a very important property because the redox balance of living cells is constantly challenged by newly formed ROS and their nitrogen analogues that can alter main macromolecules, essential for tendon structure and function [76,77]. To counteract oxidative damage, the body is endowed with protective enzymes (superoxide dismutase, catalase, among others) and a set of low molecular weight compounds capable of neutralising ROS. Curcumin and analogs support this process by scavenging oxygen species (e.g., superoxide, hydroxyl radicals, and NO) [78]. The assumption that the antioxidant activity of curcumin is related to anti-inflammatory effects is supported by the results of the inhibition of the main enzymes responsible for the transformation of arachidonic acid into prostaglandins, lipooxygenase (LOX), and COX, by natural and synthetic curcuminoids [79,80,81].

## 2. Methodology

### Study Analysis and Search Strategy

**Figure 1:**
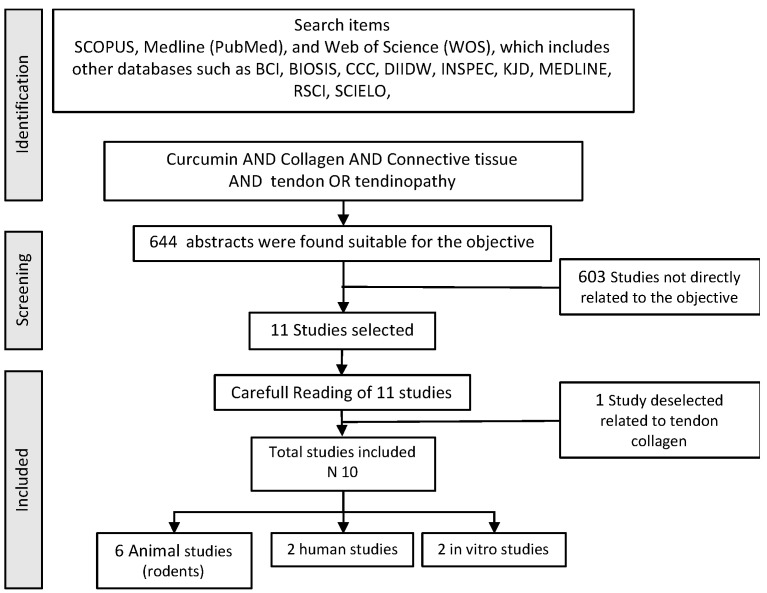
Flowchart used in the bibliographic search.

To conduct the present review, a structured search of SCOPUS, Medline (Pub-Med), and Web of Science (WOS) databases was performed. The search used the following words related to curcumin, tendons, and tendinopathy (“curcumin” AND “tendon” OR “tendinopathy” OR “tendinosis” OR “Tenocyte”). Titles and abstracts were separated from the search to identify duplicates and possibly missing articles. The appropriateness of the articles was assessed according to the GRADE concept [82] and the level of evidence [83]. All articles were selected if they had “Moderate” or “High” scientific quality and a gradable grade of evidence from 2 to 2++. Inclusion criteria included: studies that aimed to identify a beneficial effect of any form of curcumin as a sole use, or as an adjuvant to other products, either in the restoration of the tendon tissue, whether or not associated with tendinopathy, or that were randomized, double-blind controlled studies with parallel design in animal samples or in humans. The complete search strategy is shown in Figure 1.

The search was performed according to the Cochrane guidelines for systematic reviews [84]. The Preferred Reporting Items for Systematic Reviews and Meta-Analyses (PRISMA) guidelines were followed [85]. The evaluation was conducted as a Scoping review to examine the extent (size), range (variety), and nature (characteristics) of the evi-dence of the possible effect of curcumine on the tendon healing and also to determine the value of undertaking a systematic review, summarize findings from a body of knowledge that is heterogeneous in methods or discipline, or identify gaps in the literature to aid the planning and commissioning of future research [86,87]. To analyze the risk of bias, we also used Cochrane guidelines [86]. In view of the domains provided by the tool, we scored those studies satisfying four or more low-risk bias domains as low risk and the remainder as high risk. 

**Figure 2:**
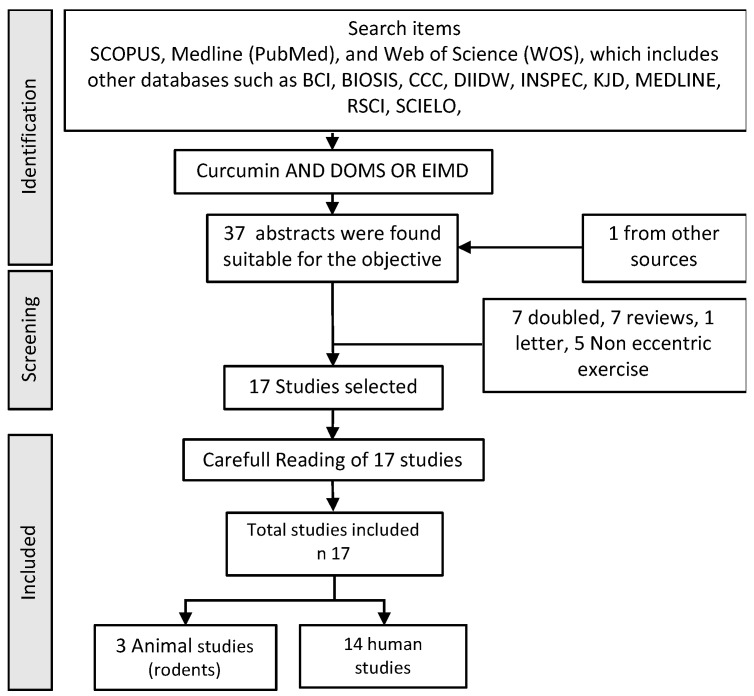
Flow chart used in the bibliographic search in humans and animals.

The final result of the search provides results which, although illustrative of the effect of the use of curcumin on the tendon, are not very homogeneous, considering the different administrations and applications in human samples or experimental animals. It has therefore been decided to offer, in addition, a list of those studies in which the use of “Curcumin” AND “Delayed Onsed Muscular Soreness” (DOMS) OR “Exercise Induced Muscular Injury” when the method of provoking the injury was through the use of eccentric work, not fatigue, not long duration exercise. The reason is that this model of injury with this provocative noxa primarily affects the myotendinous area and its connective tissue. The evaluation flow is presented below in Figure 2.

## 3. Results

**Figure 3:**
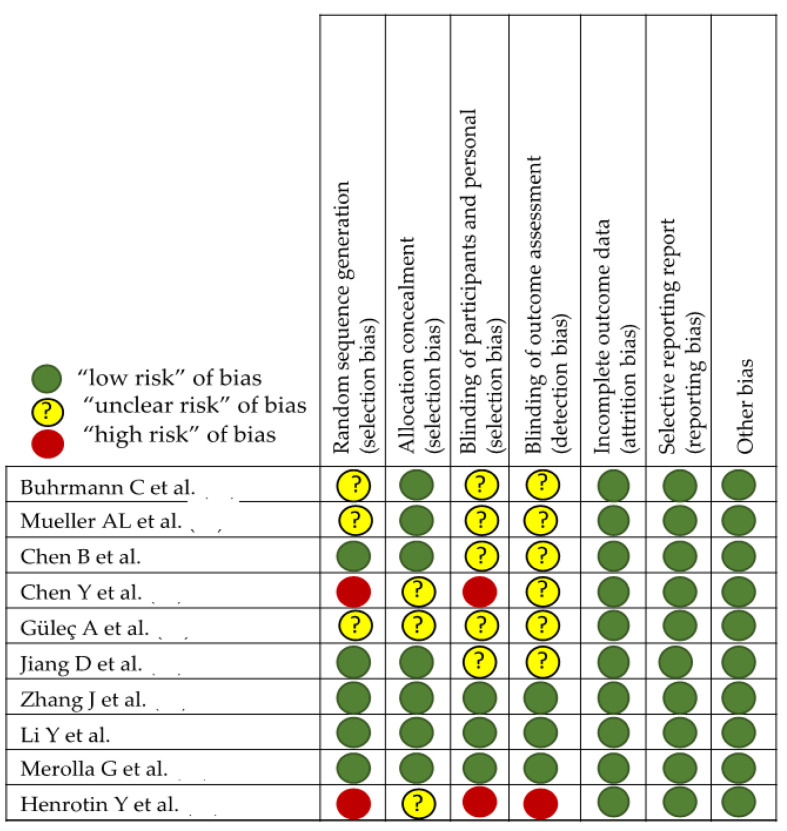
Effect of curcumin on tendinopathy healing [87,88,89,90,91,92,93,94,95,96].

**Figure 4:**
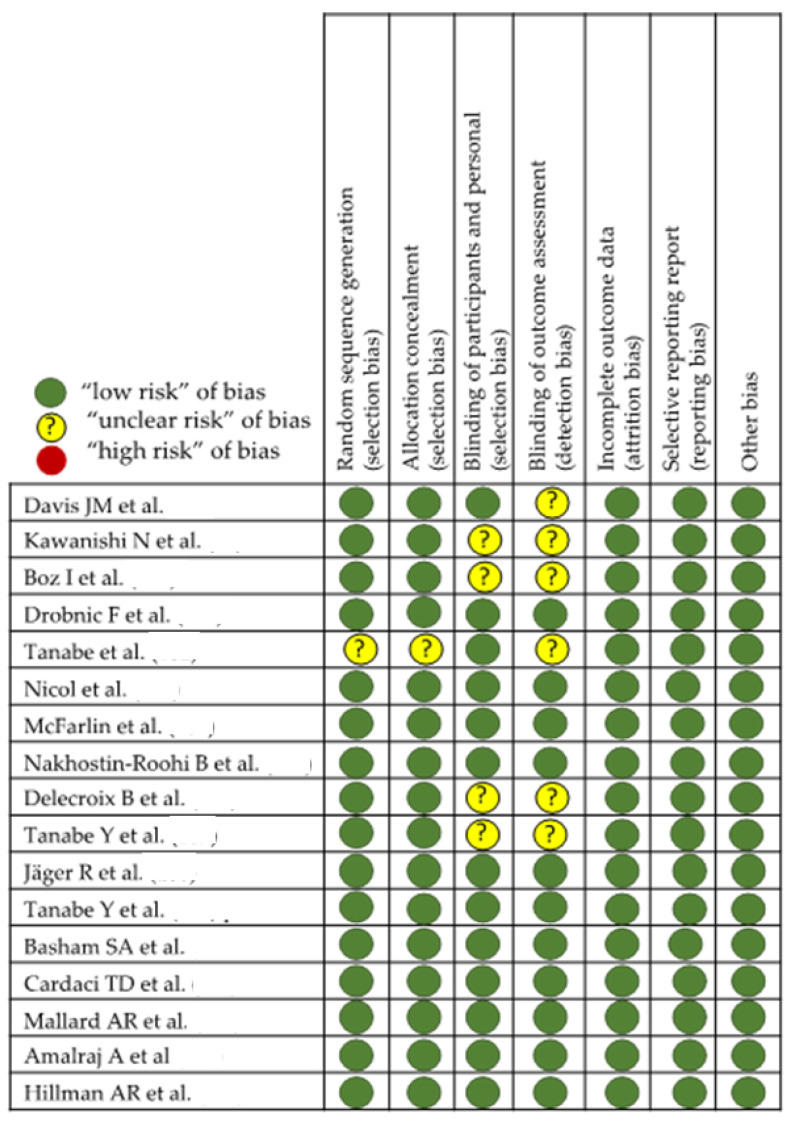
Effect of curcumin on MTJ eccentric injury [97,98,99,100,101,102,103,104,105,106,107,108,109,110,111,112,113,114].

In the evaluation of bias (Figure 3 and Figure 4), in studies with laboratory animals, it can be observed that the randomization of the animals is absent or not discussed, except in the MTJ-DOMS studies (Figure 4). Although it might not influence the study, this is unknown. Finally, except in three studies, it is common for the investigator to work blindly without knowing from which animal the sample came, but this is not indicated [88,89,90,91,92]. 

The type of molecule and whether or not there is a commercial brand, is exposed in the column “Molecule” and the administered curcumin dosage, and the route is indicated in the proper columns “dosage” and “route”. In the Tables, within the corresponding column, it is indicated whether the study had a control group and a placebo group or not. The number and type of subjects included in the study are shown in the “n and Type of subjects” column. The next two columns are related to the type of injury evaluated and the tests or procedures to study the effect. Finally, the last column exposes the “Impact on resolution” of the injury. The two upper studies [87,93] are in vitro and show a better modulation of the inflammatory and degradative processes enhancing the down-regulation of the collagen. Rodent studies [88,89,90,94,95,96] conclude that curcumin seems to promote tendon-to-bone healing associated with anti-inflammatory and antioxidation effects, with a better functional recovery. Finally, the human studies [91,92] related to surgery procedures include a better pain score, concomitant with the usual treatment, and patient satisfaction. In relation to Table 1 and Table 2, which evaluate the effect of the use of curcumin against DOMS in the MTJ, due to eccentric work, a lower presence of pain perception and lower creatine kinase (CK) values seem a common result, although there are those that do not observe changes [97,98,99,100,101,102,103,104,105,106,107,108,109,110,111,112,113,114]. The studies that determine cytokines are not conclusive and show diverse results, being better described in [100,102,103,109], although it is necessary to point out that no changes in TNF-α were reported in [101,102,109,112], while positive changes reported in [97,103,110].

When evaluating the possible effect of curcumin on the injury response of an eccentric work such as DOMS in MTJ, we pleasantly observed that randomization and double-blinding are present in almost all of them. It is assumed that the statistical study and the evaluation of the results remain with the double-blind method until the discussion begins once the study arms are identified. However, there are three of them, in this group of studies with rodents [97,98,99], in which the presence of double blinding is not feasible and the administration of the results in an unknown way is not faithfully exposed in the methodology. In general, evaluating the effect of the use of curcumin against DOMS in the MTJ, due to eccentric work, a lower presence of pain perception and lower creatine kinase (CK) values seem a common result, although there are those that do not observe changes [98,99,100,105,106,109,110,111,112,113,114]. On the other hand, there are two studies where the design is crossover [105,106] and where, despite explicitly exposing the randomization method, the presence of a double blind that reaches up to the evaluation of the results, is not well determined. The data from each of the studies are presented in Table 1, Table 2 and Table 3. Table 3 is related to studies on the effect of curcumin on tendon and Table 1 and Table 2 on MTJ injury related to eccentric work and DOMS. The content of the tables is discussed in the corresponding section. There are 10 different studies with curcumin on the tendon and 17 on MTJ.

## 4. Discussion

This review highlights the key role of curcumin in inflammatory and degradative processes by blocking the action of proinflammatory citokines such as TNF-α, and other inflammatory markers, facilitating collagen and tenocyte-specific transcription factor regulation. This leads to the conclusion that curcumin is a nutritional element that helps in promoting tendon healing through these anti-inflammatory and antioxidant effects.

### 4.1. Curcumin as Anti-Inflammatory and Immunomodulatory Agent

The pharmacological actions of curcumin as an anti-inflammatory agent have already been examined by Srimal and Dhawan [115]. Ammon et al. [116] have demonstrated that curcumin inhibits leukotriene formation in rat peritoneal polymorphonuclear neutrophils. Leukotrienes (LT) are metabolites derived from the oxidative metabolism of arachidonic acid via the 5-lipoxygenase pathway.

Also, the anti-inflammatory effect of curcumin is related to its ability to suppress acute and chronic inflammation [117]. NF-κB plays a key role in signal transduction pathways that are involved in inflammatory diseases and several cancer types [118]. Curcumin inhibits TNF-α dependent activation of NF- kβ [119]. COX-2 is present in inflammatory processes, and curcumin decreases its expression and inhibits the expression of the pro-inflammatory enzyme 5-LOX, which transforms fatty acids into leukotrienes.

Furthermore, curcumin induces down-regulation of several inflammatory cytokines such as TNF-α, IL-1β, IL-6, IL-8, interferon, and other chemokines [120,121]. In vivo studies in humans and animals, curcumin has also revealed anti-inflammatory effects. Several clinical trials revealed the efficacy of curcumin in inflammatory diseases such as inflammatory bowel disease (IBD) in humans through the modulation of inflammatory factors [122]. In the study by Chan et al. [123], curcumin supplementation (92 ng/g body weight of curcumin) was observed to have an anti-inflammatory effect by reducing iNOS mRNA expression (50–70%) in the liver of mice injected with lipopolysaccharide [123]. 

As mentioned before, curcumin has a variety of pharmacological properties that help to reduce tissue damage and promote tissue repair. Curcumin has antioxidant, anti-inflammatory, and anti-infective properties. In addition, curcumin may down-regulate NF-κβ involved in apoptosis, matrix degradation, and inflammation in tenocytes. NF-κβ helps to control many functions in the cell, such as growth and survival, as well as controlling immune and inflammatory response [74,87,124,125].

Curcumin induces down-regulation of proinflammatory interleukins (IL-1β, IL-2, IL-6, IL-8, and IL-12), inflammatory cytokines, and monocyte chemotactic protein-1 (MCP-1), by inhibiting the transcriptional signalling pathway (JAK/STAT). In addition, overexpression of B-cell lymphoma-2 (Bcl-2) or B-cell lymphoma-extra large (Bcl-xL) controls the intrinsic apoptosis pathway and protects cells against apoptosis. Otherwise, both cell types counteract proapoptotic and proinflammatory attacks and restore the physiological anti-inflammatory response [74,126].

In addition, curcumin acts as an immunomodulator factor by suppressing acquired immune responses in T-cells, inhibiting activation, differentiation, and cytokine production [127].

#### Pharmacological Action of Curcumin

- Antioxidant

Curcumin also behaves as an antioxidant showing its positive effects on cell regeneration and wound healing. However, it has negative effects on neoangiogenesis (a frequent anatomopathological finding in tendinopathies). Inhibitory activity has also been observed with respect to ECM MMPs [128].

Curcumin functions as a scavenger of oxygen species (e.g., superoxide, radical hydroxyl, and NO) [78].

Oxidative stress plays an important role in the pathogenesis of various diseases [129,130]. Curcumin has been found to be a free radical scavenger and reducing agent, resulting in the inhibition of DNA damage. In addition, in vitro studies have shown that curcumin inhibits the production of NO and ROS in macrophages [131,132]. In animals, it has also been shown to inhibit lipooxygenase and cyclooxygenase in fibroblasts [133]. In addition, curcumin could increase collagen synthesis, promote angiogenesis, decrease ROS and improve the wound healing process. An optimised COL-I/COL-III ratio could explain the optimal matrix organisation in tendon healing. Therefore, curcumin enhances tendon regeneration through well-organised collagen fibres, extensive collagen deposition, decreased ROS level, and increased biomechanical properties of regenerated tendon tissues [74,88,127,134].

Curcumin moderates neuronal damage and the neuroprotective effects are induced by N-methyl-D-aspartate (NMDA) [135]. Therefore, curcumin, acts as a neuro-protective and anti-inflammatory agent. For instance, curcumin has been considered an adjuvant in the treatment of multiple sclerosis, rheumatoid arthritis, and psoriasis [136,137,138].

- Collagen organisation

The administration of curcumin inhibits the crosslinking of collagen bundles with alteration of the molecular structure [23]. In addition, curcumin inhibits the proliferation of endothelial cells modulating basic fibroblast growth factor (bFGF) [24] and suppressing angiogenesis, a common finding in chronic tendinopathy. For all these reasons, curcumin is a key candidate for the treatment of this pathology.

As commented before, the oral administration of curcumin over a long period of time decreases the accumulation and crosslinking of collagen, returning it to its original structure and properties. On the other hand, collagen salt precipitation and altered acid digestion are common findings in tendinopathies, and curcumin administration is able to restore tendon characteristics [139].

In rats with a tendon injury, the animals treated with curcumin showed deposition of well-organised collagen fibres, with lower levels of MDA and an increase of biomechanical properties, together with an increase in the activity of manganese superoxide dismutase (Mn-SOD), mitochondrial antioxidant [88].

- Analgesic

One of the most important pathological signs of tendinopathy is pain. Tendinous pain is accompanied by muscle pain (related to the MTJ) which leads to increased muscle tone. The effect of pain and increased muscle tone hinders physical therapy and the tissue recovery process [140]. However, these findings do not always have an explanation supported by scientific evidence. This is because in humans the situation seems to be more complex than in animal models [141,142]. In this context, chronic pain tends to inhibit or make difficult the voluntary and reflex contractile activity of the affected muscle or its agonists. This fact suggests that these effects are beneficial and provide a protective adaptation, and are not only the cause of pain. In any case, the administration of analgesics will improve the quality of life and facilitate the implementation of physical therapy in the treatment of the affected tissue.

Curcumin has an effect on pain perception [143]. In this regard, several clinical trials have demonstrated the analgesic effect of curcuminoids, alone or administered with other analgesics, in various pain conditions, such as osteoarthritis, chronic postsurgical pain, and active rheumatoid arthritis [138]. The analgesic effect may be mediated by a modulation of the inflammatory and oxidative responses in muscle injury as previously explained [100,144]. Essentially, this analgesic effect is explained by curcumin’s ability to: (a) inhibit the production of PGE2 by inhibiting the expression of the COX-2 gene [145]; (b) stimulate the production of cortisol by the adrenal gland inhibiting potassium channels; (c) reducing the nerve endings of the neurotransmitter substance P [146,147] and (d) by desensitising the transient receptor potential vanilloid 1 (TRPV1) and ankyrin 1 (TRPA1) ion channels responsible for pain sensation, thereby reducing pain sensitivity [148,149]. Through these actions, curcumin can limit post-exercise inflammation, which in turn reduces pain sensitivity and DOMS. It has been suggested that curcumin promotes the modulation of TRPV1 rather than modifying inflammatory responses to exert antinociceptive effects [150,151].

- Biostimulator

Curcumin may induce pharmacokinetic alterations when it is used in combination with drugs to treat cardiovascular conditions, antidepressants, anticoagulants, antibiotics, chemotherapeutic agents, and antihistamines [152,153]. Curcumin seems to have some biostimulatory power and is used as a biopotentiator for antimicrobial agents and anticancer drugs [80,154]. The fundamental mechanism used by curcumin to carry out these actions is through the suppression of drug metabolising enzymes in the liver [155]. This must be considered when curcumin is administered in patients suffering tendinopathy and who are being treated with other drugs such as antihistamines that are, both together, well tolerated and compatible for a short time period [156]. Administration of curcumin together with antibiotics such as norfloxacin [157] or antifungals, enhanced lightly the area under the curve [158]. No apparent change in pharmacokineticsis is observed when curcumin is administered in combination with commonly used anti-inflammatory or antalgesic drugs. However, as indicated by Fredriksson [159], NSAIDs (non-steroidal anti-inflammatory drugs) may affect the healing of the tissues of the musculoskeletal system, and in particular the proliferation of tenocytes after injury. Nevertheless, the scientific literature does not provide sufficient evidence for or against the use of NSAIDs following acute injury or surgical repair of the tendon-bone interface [160,161,162]. Despite this controversy, the use of NSAIDs in chronic musculoskeletal pathology, particularly tendinopathies, is common. The use of curcumin in association with NSAIDs during prolonged inflammatory processes can be synergistic and further investigated [163,164].

### 4.2. Therapeutic Guidance

At present, it is not easy to define a suitable dose of curcumin for therapeutic purposes. As observed in Table 3, there is no homogeneity about what might be called the optimal dose and therapeutic use. Unformulated curcumin, administered orally, is poorly bioavailable in humans due to its low digestive absorption, protein binding, rapid metabolism, and systemic elimination from the body [165,166]. Some pharmaceutical formulations or certain combinations with other compounds, increase the solubility, prolong plasmatic presence and improve the pharmacokinetic profile and cellular uptake [167,168,169].

To optimise the bioavailability of curcumin, new delivery systems have been developed, such as the solid lipid particle, the micellar system, or hydrophilic nanoparticles, which are able to increase the concentration of curcumin by up to 15–20 times [170,171]. Therefore, giving an indication from the different studies for an anti-inflammatory dose is somewhat arbitrary. The absence of a recommended dose is based in the variability coming from different formulations used in the different studies performed in tendons and DOMS. They present different bioavailability and not all studies determine the plasma level, in order to associate the possible therapeutic effect with a given plasma concentration. 

However, despite low serum levels after oral administration of curcumin, beneficial effects in vivo are evident [79]. Based on the studies evaluated in this review, possible effective doses range from 90 mg in bioavailable compounds to 5 g in *Curcuma longa* extracts [172]. An appropriate therapeutic dose for curcumin would be between 1–1.5 g, spread over 2–3 daily doses, to maintain more sustained serum levels, considering that plasma concentrations of curcumin peak 1–2 h after ingestion and gradually decline over 12 h [173,174,175,176,177,178]. 

## 5. Conclusions

Curcumin is a product that has demonstrated consistent biological analgesic, anti-inflammatory, and antioxidant properties. Supplementation has a positive effect on the inflammatory and regenerative response in tendinopathy. Curcumin decreases and modulates the infiltration, activation, and maturation of leukocytes, as well as the production of pro-inflammatory mediators at the site of inflammation. Curcumin inhibits angiogenesis at the injured site and decreases the accumulation and crosslinking of collagen, returning it to its original structure and properties. Considering the ability of curcumin to facilitate the action of other drugs, its wide therapeutic margin with minimal or no unwanted side effects makes it a promising molecule for therapeutic use in isolation or as a complement to other pharmacological therapies in musculoskeletal pathology, particularly tendinopathies.

## Figures and Tables

**Table 1:** Studies evaluating the effect of curcumin in miotendinous junction related to DOMS.

Reference		Molecule/s	Dosage	Route	Days	Placebo/Control	n	Type of Subjects	Type Injury	Tests	Impact on Resolution.
Davis JM et al. (2007)	[99]	Curcumin	10 mg (≈40 mg/kg)	Oral (pellets)	3	P	64	Rodents	Eccentric exercise or Concentric exercise	Blood analysis (CK, Inflamation), FP	⊕ Running time to fatigue, running distance, IL-1β, IL-6, TNF-α, in the eccentric exercise working set of animals.
Kawanishi N et al. (2013)	[100]	Curcumin (Theracurmin^®^)	3 mg	Oral (after injury)	1	P	52	Rodents	Eccentric exercise	Blood analysis (M Injury, Inflamation pattern) muscle immunohisyology	⊕ NADPH oxidase, MCP-1 and F4/80 mRNA expression, H_2_O_2_, attenuated oxidative stress ≈ CK, LDH, COX2 expression.,
Boz I et al. (2014)	[101]	Curcumin	200 mg/kg	Oral gavage	20	P	30	Rodents	Eccentric exercise	Blood analysis (M Injury, Inflamation pattern, oxidative pattern) Muscle histology,.	⊕ CK, Mb ≈ MDA, SOD, GPx
Drobnic F et al. (2014)	[102]	Curcumin (Meriva^®^)	200 mg/day (2 g Meriva^®^)	Oral	4	P	20	Human	Eccentric exercise	Blood analysis (CK, Inflamation, oxidative pattern), VAS.	⊕ IL-8, VAS; ≈ CRP, MCP1, CK, TAC.
Tanabe et al. (2015)	[103]	Curcumin (Theracurmin^®^)	150 mg/day	Oral	1	P	14	Human	Eccentric exercise	Blood analysis (M Injury, Inflamation pattern), VAS, FP, Arm circumference	⊕ CK, MVIC ≈ ROM, VAS, IL-6, TNF-α, Arm circumference
Nicol et al. (2015)	[104]	Curcumin (Eurofins Scientific Inc)	2.5 g/12 h	Oral	5	P	19	Human	Eccentric exercise	Blood analysis (M Injury, Inflamation pattern), VAS, FP	⊕ VAS, CK, IL-6, Exercise performance. ≈ Swelling, TNF-α
McFarlin et al. (2016)	[105]	Curcumin (Longvida^®^)	400 mg/day	Oral	6	P	28	Human	Eccentric exercise	Blood analysis (CK, Inflamation, oxidative pattern), VAS, FP	⊕, CK, IL-8, TNF-α. ≈ VAS, IL-6, IL-10
Nakhostin-Roohi B et al. (2016)	[106]	Curcumin (Theracurmin^®^)	1150 mg	Oral (after injury)	1	P	10	Human	Eccentric exercise	Blood analysis (M Injury, Inflamation pattern), VAS.	⊕ VAS, CK, ALT, AST, TAC
Delecroix B et al. (2017)	[107]	Curcumin + Piperine (*)	2 g/8 h	Oral	4	P	10	Human	Eccentric exercise	Blood analysis (M Injury, Inflamation, oxidative pattern), VAS, FP	⊕ Exercise performance. ≈ VAS, CK.
Tanabe Y et al. (2019)	[108]	Curcumin (Theracurmin^®^)	90 mg/12 h	Oral	11	P	24	Human	Eccentric exercise	Blood analysis (CK), VAS, FP	⊕ VAS, ROM ≈ MVIC, CK.

⊕: Effective; ≈: Ineffective; (*): Placebo added 20 mg of piperine as the curcumin treatment; AST: Aspartate aminotransferase; BAP: Biological antioxidant power; CAT: Catalase; CK: Creatine kinase; CLE: *Curcuma longa* extract; CRP: C-Reactive protein; d: Day; d-ROMs: Diacron-reactive oxygen metabolites; ESR: Erythrocyte sedimentation rate; FRAP: Ferric reducing ability plasma; GPx: Glutathione peroxidase; Hs: High sensitivity; IL-6: Interleukin-6; IL-8: Interleukin-8; IL-10: Interleukin-10; La: Lactate; LDH: Lactate dehydrogenase; Mb: Myoglobin; MCP-1: Monocyte chemoattractant protein 1; MVIC: Maximal voluntary isometric contraction; P: Placebo; ROM: Range of motion; TC: Tight circumference; TNF-α: Tumor necrosis factor-α; UPS: Ubiquitin proteasome system; VAS: Visual analogue scale; VO_2_ max: Maximal oxygen consumption.

**Table 2:** Studies evaluating the effect of curcumin in miotendinous junction related to DOMS.

Reference		Molecule/s	Dosage	Route	Days	Placebo/Control	n	Type of Subjects	Type Injury	Tests	Impact on Resolution.
Jäger R et al. (2019)	[109]	Curcumin (CurcuWIN^®^)	50 or 200 mg/day	Oral	56	P	74	Humans	Eccentric exercise	VAS, FP (Isokinetic and isometric test)	≈ VAS, isokinetic strength tests. ⊕ tendency in VAS and some isokinetic test for the 200 mg dosage.
Tanabe Y et al. (2019) Ex I	[110]	Curcumin (Theracurmin^®^)	90 mg/12 h	Oral Before injury	7	P	10	Human	Eccentric exercise	Blood analysis (M Injury, Inflamation, oxidative pattern), VAS, FP	⊕ IL-8, ≈ VAS, Swelling, CK, MVIC, ROM, TNF-α, BAP,
Tanabe Y et al. (2019) Ex II				Oral After injury			10				⊕ VAS, CK, MVIC, ROM, d-ROMs. ≈ Swelling, IL-8,, TNF-α, BAP, d-ROMs.
Basham SA et al. (2020)	[111]	Curcumin (CurcuFresh^®^)	1.5 g/day	Oral	28	P	20	Human	Eccentric exercise	Blood analysis (M Injury, Inflamation pattern, oxidative pattern), VAS	⊕ CK, VAS, TAC, MDA, TNFα,
Cardaci TD et al. (2020)	[113]	Curcumin + Piperine (*)	2 g/day	Oral	11	P (Piperine)	23	Human	Eccentric exercise	Muscle biopsy, VAS,	⊕ Ubiquitin, MAFbx/Atrogin-1. UPS dysregulation is mediated by downregulation of proteasome catalytic enzymes. ≈ USP19, VAS.
Mallard AR et al. (2020)	[114]	Curcumin (+LipiSperse)	450 mg/day	Oral	1	P	27	Human	Eccentric exercise	Blood analysis (CK, Inflamation, oxidative pattern), VAS, FP	⊕, VAS, Swelling (TC), Lac, IL-10, IL-6, IL-8, ≈ CK, LDH, CRP-Hs, Mb, TNF-α,
Amalraj A et al. (2020)	[115]	Curcumin (Cureit^®^)	500 mg/day	Oral	4	P	30	Human	Eccentric exercise	Blood analysis (CK), VAS, FP (maximal aerobic exercise testing)	⊕ VAS, ≈ CK, VO_2_ max
Hillman AR et al. (2021)	[116]	Curcumin (CLE)	475 mg/day	Oral	10	P	22	Humans	Eccentric exercise	Blood analysis (CK, ESRVAS, FP (vertical jump)	⊕ VAS, Vertical jump ≈ CK, ESR

⊕: Effective; ≈: Ineffective; (*): Placebo added 20 mg of piperine as the curcumin treatment.; AST: Aspartate aminotransferase; BAP: Biological antioxidant power; CAT: Catalase; CK: Creatine kinase; CLE: *Curcuma longa* extract; CRP: C-Reactive protein; d: Day; d-ROMs: Diacron-reactive oxygen metabolites; ESR: Erythrocyte sedimentation rate; FRAP: Ferric reducing ability plasma; GPx: Glutathione peroxidase; Hs: High sensitivity; IL-6: Interleukin-6; IL-8: Interleukin-8; IL-10: Interleukin-10; La: Lactate; LDH: Lactate dehydrogenase; Mb: Myoglobin; MCP-1: Monocyte chemoattractant protein 1; MVIC: Maximal voluntary isometric contraction; P: Placebo; ROM: range of motion; SOD: Superoxide dismutase; TC: Tight circumference; TNF-α: Tumor necrosis factor-α; UPS: Ubiquitin proteasome system; VAS: Visual analogue scale; VO_2_ max: Maximal oxygen consumption.

**Table 3:** Studies evaluating the effect of curcumin in tendinopathy.

Reference		Molecule/s	Dosage	Route	Days	Placebo/Control	n	Type of Subjects	Type Injury	Tests	Impact on Resolution.
Buhrmann et al. (2011)	[94]	Curcumin	5–20 μM	Tenocytes culture in vitro	12 h, 24 h, 48 h	C	-	Human tenocytes monolayer cultures	IL-1β stimulated tenocytes	Histology, Immunology	⊕ modulation of NF-κB signaling, inhibited IL-1β-induced inflammation and apoptosis. Any toxicity on the cells
Mueller AL et al. (2022)	[95]	Calebin A	5000 μM DMSO (<0.1%)).	Tenocytes culture in vitro	18 h	C	-	Canine tenocytes monolayer cultures	Tendinitis μenvironment	Histology, Immunology	⊕ inflammatory and degradative processes by blocking TNF-β, TNF-α-induced adhesiveness and T-lymphocytes facilitating down-regulation of Collagen I, Tenomodulin, tenocyte-specific transcription factor and the up-regulation of NF-κB phosphorylation;.
Chen B et al. (2021)	[96]	Curcumin, Mg, Chitosan		Hydrogel locally applied during surgery	3–4–21	C		Rodents	Rotator cuff C/R	Histology, Immunology, Biomechanical.	Promote rotator cuff tendon-to-bone healing ⊕ anti-inflammatory and antioxidation effects, ⊕ biomechanical tests and histological results
Chen Y et al. (2019)	[88]	Curcumin, Chitosan	135 mg/mL	Hydrogel locally applied during surgery	Every 3 d/4 weeks	C	21	Rodents	Achilles tendon ectopic calcification model	Imaging (xR), Histology, Immunology.	⊕ Partially reversed tendon calcification and enhanced tendon regeneration
Güleç A et al. (2018)	[89]	Curcumin	200 mg/kg	Oral gavage	28	C	18	Rodents	Achilles tendon C/R	Histology and Biomechanical	⊕ Tenocyte morphology, collagen, and ground substance scores, ⊕ Biomechanical parameters (failure load, cross-sectional area, length, ultimate stress, strain), ↔ Vascularization,
Jiang D et al. (2016)	[90]	Curcumin	100 mg/kg	Oral gavage	14	C	64	Rodents	Patellar tendon	Histology, Immunology Gene expression, Biomechanical	⊕ Organized collagen fiber, alignment, ⊕ collagen I, ⊕ biomechanical properties and ↑SOD, ↓MDA.
Zhang J et al. (2021)	[91]	Curcumin Celecoxib	-	Electrospun polyester membrane (EPM)	3	C	40	Rodents	Achilles tendon C/R	Histology, Immunology	The EPM with Curcumin and Celecoxib acts synergistically preventing peritendinous adhesion and inflammation.
Li Y et al. (2016)	[92]	Curcumin	0.44 mg/kg/0.1 mL saline	Polymeric nanomicelles applied locally	28	C	36	Rodents	Achilles tendon C/R	Histology, Biomechanical	⊕ inflammatory adhesion, ⊕ collagen fiber orientation ⊕ tendon strength
Merolla et al. (2015)	[97]	Curcuma, MSM, Boswellia, Vit C, Glucosamin, CS, Collagen, Arginin	200 mg	Oral	168	P	50	Human	Rotator cuff surgery	VAS, PGAS, Constant–Murley Score, Biomechanical.	⊕ postoperative rotator cuff short and partially mid-term pain, while longterm pain was unchanged, ⊕ concomitant treatment
Henrotin Y et al. (2021)	[98]	Curcumin, Boswellia	144 mg	Oral	30	No	670	Human	Diverse tendinopathies	Analog visual scale (VAS), patien satisfaction	⊕ pain score, concomitant treatment and patient satisfaction

⊕: Effective; Ineffective; ↑: Higher or improved; ↓: Lower; ↔: Similar than the Control group; CS: Condroitin sulfate; d: Days; DMSO: Dimethyl-sulfoxide; EPM: Electrospun polyester membrane; IL-1β: Interleukin-1β; MSM: C/R: cut and repair; NF-κB: Nuclear Factor-κB; PGAS: Patient Global Assessment scores; VAS: Visula analog scale; Vit C: Vitamin C.

## Data Availability

Not applicable.
